# Serotype Distribution and Antimicrobial Resistance of *Streptococcus pneumoniae* Isolates Causing Invasive Diseases from Shenzhen Children’s Hospital

**DOI:** 10.1371/journal.pone.0067507

**Published:** 2013-06-28

**Authors:** Xiang Ma, Ruizhen Zhao, Zhuoya Ma, Kaihu Yao, Sangjie Yu, Yuejie Zheng, Yonghong Yang

**Affiliations:** 1 Key Laboratory of Major Diseases in Children and National Key Discipline of Pediatrics (Capital Medical University), Ministry of Education, Beijing Pediatrics Research Institute, Beijing Children’s Hospital Affiliated to Capital Medical University, Beijing, China; 2 Laboratory of Bacteriology and Department of Respiratory Diseases, Shenzhen Children’s Hospital, Shenzhen, China; University of Malaya, Malaysia

## Abstract

**Objective:**

To provide guidance for clinical disease prevention and treatment, this study examined the epidemiology, antibiotic susceptibility, and serotype distribution of *Streptococcus pneumoniae* (*S. pneumoniae)* associated with invasive pneumococcal diseases (IPDs) among children less than 14 years of age in Shenzhen, China.

**Materials and Methods:**

All the clinical strains were isolated from children less than 14 years old from January 2009 to August 2012. The serotypes and antibiotic resistance of strains of *S. pneumoniae* were determined using the capsular swelling method and the E-test.

**Results:**

A total of 89 strains were isolated and 87 isolates were included. The five prevailing serotypes were 19F (28.7%), 14 (16.1%), 23F (11.5%), 19A (9.2%) and 6B (6.9%). The most common sequence types (ST) were ST271 (21.8%), ST876 (18.4%), ST320 (8.0%) and ST81 (6.9%) which were mainly related to 19F, 14, 19A and 23F, respectively. The potential coverage by 7-, 10-, and 13-valent pneumococcal conjugate vaccine were 77.0%, 77.0%, and 89.7%, respectively. Among the 87 isolates investigated, 11.5% were resistant to penicillin, and for meningitis isolates, the resistance rate was 100%. Multi-drug resistance (MDR) was exhibited by 49 (56.3%) isolates. Eighty-four isolates were resistance to erythromycin, among which, 56 (66.7%) carried the ermB gene alone and 28 (33.3%) expressed both the ermB and mefA/E genes.

**Conclusions:**

The potential coverage of PCV13 is higher than PCV7 and PCV10 because high rates of serotypes 19A and 6A in Shenzhen. The clinical treatment of IPD needs a higher drug concentration of antibiotics. Continued surveillance of the antimicrobial susceptibility and serotypes distribution of IPD isolates may be necessary.

## Introduction

*Streptococcus pneumoniae* (*S. pneumoniae*) is one of the most common causative pathogens of severe invasive infections (e.g., meningitis and sepsis) among infants and young children. The morbidity and mortality rates of IPD worldwide are high, particularly in developing countries. The World Health Organization (WHO) estimated that the annual incidence of IPD ranges from 10 to 100 cases per 100 000 population in Europe and the United States. In developing countries, the incidence of IPD in children aged <5 years is several times higher than it is in industrialized countries [1]. A surveillance study in Asia estimated the mean incidence of pneumococcal meningitis in children aged 1 to 23 months at 5.1 cases per 100 000 childbirths from 1999 to 2003 [2]. More recently, Chinese data showed that there were 12 815 cases/100 000/year of all-cause pneumonia among children between 1 month and 59 months, with 526 deaths/100 000 annually and there were 14 meningitis cases/100 000/year between 1980 and 2008 [3]. However, data from IPD cases are rare in many parts of Asia because of the low culture rate caused by the inappropriate use of antibiotics [4], [5], [6] and difficulties in culturing *S. pneumoniae* in vitro [7].

This situation has been worsened by the rapid spread of antimicrobial-resistant *S. pneumoniae*. Antibiotic-resistant pneumococci have been increasing since the 1990s and are becoming a major problem worldwide. The emergence of multidrug-resistant *S. pneumoniae* (MDRSP) has been observed in various countries over the past decades. The growing resistance of *S. pneumoniae* to commonly used antibiotics underlines the urgent need for vaccines to be used to control pneumococcal disease.

There are two types of *S. pneumoniae* vaccine available that can be classified as polysaccharide vaccine and glycoconjugated polysaccharide vaccine. At present, the 7-valent glycoconjugated vaccine (PCV7, which included pneumococcal serotypes 4, 6B, 9V, 14, 18C, 19F, and 23F) has been regularly used for all infants and children aged two to 59 months who are at increased risk for pneumococcal disease in Europe and the United States [8]. Since the introduction of PCV7, it had showed high effectiveness in controlling IPD [9]. However, the coverage of PCV7 varies with the different population being vaccinated and may range from 60% in Asia to 70% to 85% in the United States and Europe [1]. At present, two new vaccines based on PCV7, PCV10 (including additional serotypes 1, 5 and 7F to PCV7) and PCV13 (including additional serotypes 1, 3, 5, 6A, 7F and 19A to PCV7) had been used in some developed countries [1]. To be effective in controlling pneumococcal disease, the composition of vaccine must match as closely as possible the prevalence of serotypes in each region. Therefore, it is necessary to monitor pneumococcal serotypes at local regions for the design of effective vaccine formulations.

The current study is conducted to examine the serotype distribution and antimicrobial resistance pattern of *S. pneumoniae* in Shenzhen, a city located in the south of China, for guiding clinical usage of antimicrobial agents and application of pneumococcal vaccines in public health control programs.

## Materials and Methods

### 2.1 Clinical Isolates

The study protocol was in accordance with the ethical standards of the responsible regional committee on human experimentation and the Helsinki Declaration of 1975 (as revised in 1983). It was approved by the ethics committee of Shenzhen Children’s Hospital.

A total of 89 isolates were identified as *S. pneumoniae* during a clinical work in Shenzhen Children’s Hospital from January 2009 to August 2012. All presumptive *S. pneumoniae* isolates were delivered to the Laboratory of Microbiology and Immunology, Beijing Children’s Hospital, Beijing, China. The isolates were identified based on their alpha-hemolytic colony, alpha hemolytic, and Gram positive diplococci. The identification was confirmed by optochin sensitivity test (Oxoid, Basingstoke, Britain), bile solubility test, and Omni serum assay (Statens Serum Institute, Copenhagen, Denmark). Only 87 of these isolates were covered in this study because of the loss of two isolates from the blood sample collected in 2012.

Among the 87 isolates, 24 were collected from 2009, 32 from 2010, 23 from 2011, and eight from 2012. In addition, 71 (81.6%) strains were isolated from blood samples, nine (10.4%) from cerebral spinal fluid samples, three (3.4%) from abscess samples, and one (4.6%) each from pleural fluid, joint cavity fluid, abdominal fluid, and broncho-alveolar lavage samples. Most of these strains (78/87, 89.7%) were isolated from children aged <5 years and nine (10.3%) from children aged ≥5 years.

*S. pneumoniae* strains isolated from normal sterile specimens from children aged <14 years were collected. All isolates were stored at −80°C in a fat-free milk preservation medium until further analysis. When two isolates express the same serotype from the same subject, only one isolate was included. All isolates were typed by a capsule-quelling test using type-specific antisera (Statens Serum Institute, Copenhagen, Denmark) against the serotypes present in the 23-valent pneumococcal polysaccharide vaccine (1, 2, 3, 4, 5, 6B, 7F, 8, 9N, 9V, 10A, 11A, 12F, 14, 15B, 17F, 18C, 19A, 19F, 20, 22F, 23F, and 33F) and serotype 6A. Typing was conducted by phase-contrast microscopy according to the published procedure [10]. Strains that could not be typed using the abovementioned method were denoted as un-typed strains.

### 2.2 Antimicrobial Susceptibility Testing

The minimum inhibitory concentrations (MICs) for penicillin, amoxicillin–clavulanic acid, cefaclor, ceftriaxone, cefuroxime, erythromycin, azithromycin, sulfamethoxazole–trimethoprim (SXT), vancomycin, tetracycline, chloramphenicol, levofloxacin, and imipenem were determined using E-test strips (AB Biodisk, Solna, Sweden) [11]. Breakpoints were based on the 2010 criteria of the Clinical and Laboratory Standards Institute (CLSI) [12]. *S. pneumoniae* ATCC 49619 was used as the quality control. Isolates not susceptible to three or more classes of antimicrobials were considered MDRSP.

### 2.3 Detection of Macrolide Resistance Genes

The *ermB* and *mefA/E* resistance genes were amplified by polymerase chain reaction (PCR) for all erythromycin-resistant strains using the primers and PCR conditions as previously described [13]. Each PCR reaction contained 500 ng of template DNA, 50 mM potassium chloride, 10 mM Tris–hydrochloride (pH 8.3), 200 µM of each deoxynucleotide triphosphate, 2.5 U *Taq* DNA polymerase (Takara Bio, Dalian, China), 1.5 mM magnesium chloride, and 1.5 µM of each primer. The PCR products were visualized by 1.5% agarose gel electrophoresis and gold-view staining.

### 2.4 Multilocus Sequence Typing (MLST)

Internal fragments ∼450 bp long from the *aroE*, *gdh*, *gki*, *recP*, *spi*, *xpt*, and *ddl* genes were amplified by PCR as previously described [14]. All the STs absent in the pneumococcal MLST database were submitted to the MLST *S. pneumoniae* database for designation. The eBURST algorithm (http://eburst.mlst.net) was used to estimate the relationships among isolates. STs that share six identical alleles of the seven MLST loci with another ST in the group were subdivided into one group as a clone complex (CC).

### 2.5 Statistical Methods

All data were analyzed using WHONET software version 5.6 (WHO). The χ^2^ test, calculated using SPSS version 10.0 (SPSS Inc, Chicago, USA), was used for statistical comparisons. A two-tailed cutoff of P<0.05 was considered statistically significant.

## Results

### 3.1 Distribution of Serotypes and Coverage of PCVs

**Figure 1:**
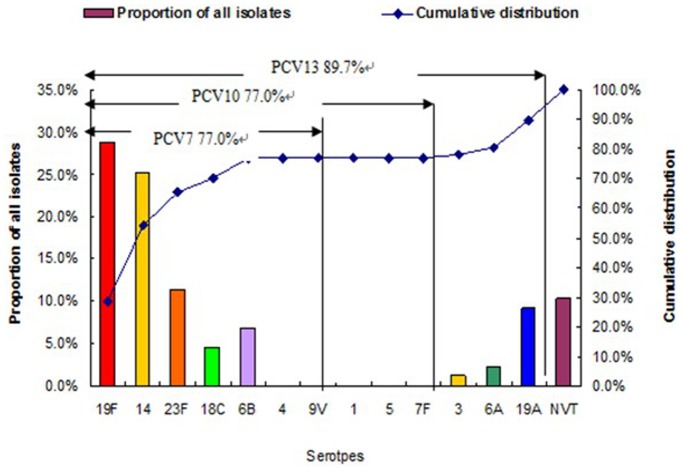
Proportionate and cumulative serotype distributions of 87 *S. pneumoniae* isolates causing invasive infections among children aged <14 years in Shenzhen Children’s Hospital from 2009 to 2012. NVT, non-vaccine serotypes not included in PCV13.

**Figure 2:**
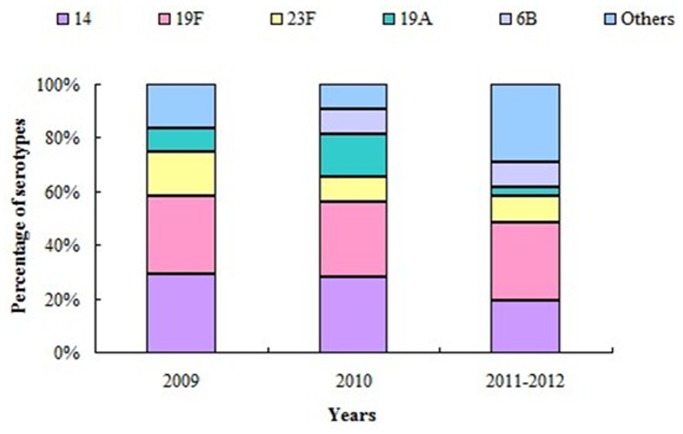
Frequency of common serotypes among the different years in Shenzhen Children’s Hospital. (For serotype 14: χ^2^ = 0.9, 19F: χ^2^ = 0.01, 19A: χ^2^ = 2.94, 23F: χ^2^ = 0.87, 6B: χ^2^ = 0.71; all P value >0.05.).

The serotype distribution of the 87 *S. pneumoniae* isolates is shown in Figure 1. Among the 87 isolates, 19F (n = 25, 28.7%), 14 (n = 22, 25.3%), 23F (n = 10, 11.5%), 19A (n = 8, 9.2%), and 6B (n = 6, 6.9%) were the most common serotypes. These five serotypes accounted for 81.6% of all the isolates. Two strains (2.3%) were un-typed. The overall coverage rates of PCV-7, PCV-10, and PCV-13 were 77.0%, 77.0%, and 89.7%, respectively (Figure 1). No significant differences in the distribution of the common serotypes were observed among the different years (Figure 2).

### 3.2 Antimicrobial Susceptibility Testing

The antibiotic activities of the 87 *S. pneumoniae* isolates against the 13 antimicrobials are presented in Table 1. According to the revised CLSI breakpoints for parenteral penicillin (resistant ≥8 µg/mL for non-meningitis isolates and ≥0.12 µg/mL for meningitis isolates), the prevalence rates of penicillin resistance were 1.3% and 100% in the non-meningitis and meningitis isolates, respectively. The proportion of isolates resistant to ceftriaxone was 3.8% for the non-meningitis and 33.3%meningitis isolates. The cefuroxime and cefaclor resistance rates were 79.3% and 81.6%, respectively. All the isolates were susceptible to vancomycin, amoxicillin–clavulanic acid, and levofloxacin, except for one (1.1%) isolate that was resistant to levofloxacin. Most of these isolates showed high resistance to macrolide antimicrobials, whereby 84 (96.6%) were resistant to both erythromycin and azithromycin with an MIC of >256 µg/mL. The non-susceptibility rates to imipenem, tetracycline, and SXT were 72.9%, 94.3%, and 55.1%, respectively.

**Table 1:** Susceptibility and minimum inhibitory concentrations of 13 antimicrobials for the 87 *S. pneumoniae* isolates.

Antimicrobials	No of isolates	Susceptibility			MIC(µg/ml)		
		Resistant	Intermediate	Susceptible	50%	90%	Range
Penicillin							
Meningitis	9	9(100%)	0	0	1	2	0.125–2
Non-meningitis	78	1(1.3%)	2(2.6%)	75(96.1%)	1	2	0.016–8
Amoxicillin–clavulanic acid	87	0	5(5.7%)	82(94.3%)	0.75	2	0.023–2
Cefuroxime	87	69(79.3%)	3(3.4%)	15(17.2%)	3	6	0.016–24
Ceftriaxone							
Meningitis	9	3(33.3%)	5(55.6%)	1(11.1%)	1.5	2	0.032–2
Non-meningitis	78	3(3.8%)	26(33.3%)	49(62.9%)	1	2	0.016–8
Cefaclor	87	71(81.6%)	2(2.3%)	14(16.1%)	32	256	0.19–512
Erythromycin	87	84(96.6%)	1(1.1%)	2(2.3%)	>256	>256	0.094–512
Azithromycin	87	85(97.7%)	2(2.3%)	0	>256	>256	1–512
Vancomycin	87	0	0	87(100%)	0.5	0.75	0.38–0.75
Levofloxacin	87	0	1(1.1%)	86(98.9%)	0.75	1	0.38–4
Imipenem	87	0	63(72.4%)	24(27.6%)	0.19	0.38	0.19–0.5
Tetracycline	87	65(74.7%)	17(19.6%)	5(5.7%)	12	24	0.094–48
Chloramphenicol	87	13(14.9%)	0	74(85.1%)	3	16	1.5–256
Sulfamethoxazole–trimethoprim	87	31(35.6%)	17(19.5%)	39(44.9%)	4	32	0.064–32

The overall rate of MDRSP was 56.3% (n = 49) (55.1% and 66.7% in the non-meningitis and meningitis isolates, respectively). The most common pattern of MDR was resistance to β-lactam antibiotics, macrolides, and SXT (n = 38, 77.6%), followed by resistance to β-lactam antibiotics, macrolides, SXT and chloramphenicol (n = 5, 10.2%). All strains with MDR were resistant to at least one of the macrolides tested. Among the 49 MDR isolates, 19F (n = 22/25, 88%), 23F (n = 10/10, 100%), 19A (n = 5/8, 62.5%), 14 (n = 4/22, 18.2%), and 18C (n = 3/4, 75%) were the most common serotypes.

### 3.3 Detection of Macrolide Resistance Genes

A total of 84 isolates of erythromycin-resistant *S. pneumoniae* were examined for macrolide-resistance genes. There were 56 (66.7%) isolates were only positive for *ermB*, but negative for *mefA*. Twenty-eight (33.3%) pneumococcal isolates contained both *ermB* and *mefA*/E. Most of (n = 27, 96.4%) the 28 isolates that carried both the *ermB* and *mefA*/E genes were typed into serotypes 19F (n = 21) and 19A (n = 6).

### 3.4 MLST

**Figure 3:**
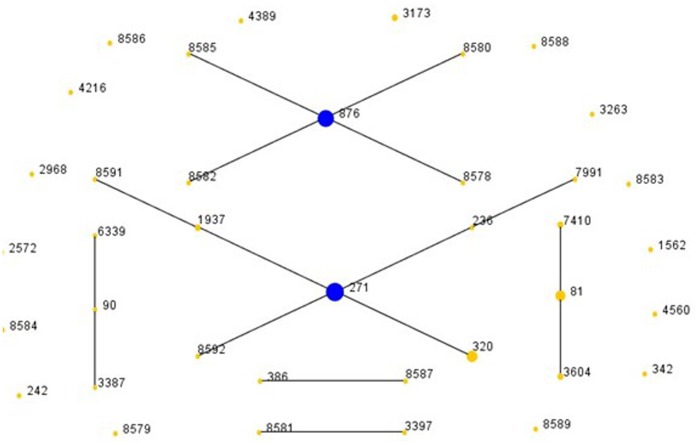
Population snapshot of the 87 ***S. pneumoniae*** strains through eBURST analysis. One spot indicates one ST. The size of one spot corresponds to the number of pneumococcal isolates with the same ST. The lines indicate the presence of single locus variant (SLV) links among particular STs.

A total of 38 STs were detected in the 87 *S. pneumoniae* isolates, of which 17 were newly assigned (2572, 6339, 8577 to 8589, 8591 and 8592) via MLST analysis. All of the new STs were novel combinations of known alleles. The four predominant STs for all pneumococci were ST271 (22.9%, 20/87), ST876 (18.4%, 16/87), ST320 (8.0%, 7/87), and ST81 (6.9%, 6/87; Figure 3), which were mainly related to serotypes 19F, 14, 19A, and 23F, respectively. Moreover, all the isolates with ST320 were typed into 19A, and the strains with ST876 were typed to serotype 14. The eBURST analysis showed six CCs and 17 singletons (Figure 3). Among all the isolates, CC271 was the most frequent CC, with a proportion of 35.6% (31/87), followed by CC876, with a proportion of 22.9% (20/87).

## Discussion

The serotypes distribution of *S. pneumoniae* reportedly varied with age, geography, diseases, and time. In this study, the most prevalent serotypes were different from previous report in either ranking order or the proportion of the predominant serotypes. Throughout Europe, the most common serotypes causing IPD were 14, 6B, 19F, and 23F. However, since the introduction of PCV7, serotypes 1, 3, 6A, 7F, and 19A have become the leading cause among the IPD isolates in several European countries [15]. Among the 115 invasive *S. pneumoniae* isolates from Thai children aged <5 years, 6B, 23F, 14, and 19F are the most common serotypes, accounting for 68% of all isolates [16]. Our colleges recently reported that the most common serotypes of IPD isolates in Chinese pediatric patients were 19F, 14, 19A, 6B, and 23F [17]. However, we found that the rank order of serotypes was 19F, 14, 23F, 19A, and 6B. Notably, eight (9.2%) of the isolates expressing serotype 19A, which were not found in the study between 2006 and 2008 [17], were found in the present study. Xue et al [17] indicated that serotype 19A is very common in northern cities but is rarely found in southern and eastern cities, including Shenzhen. This change has two potential causes. First, serotype 19A originated from Northern to Southern China. Second, 19A, as an MDR serotype, was selected under the pressure of antibiotics and easier to be transferred. As reported previously [17], PCV7 is not responsible for this increasing trend because its immunization rate is less than 1% in this city for the past four years. These data indicated that the prevailing serotypes vary in different regions and time worldwide. So, the continuous surveillance for IPD isolates, especially MDRSP such as 19A, 19F, and 23F was necessary.

PCV7, the first conjugate vaccine for preventing pneumococcal infections for children, was introduced into the routine infant immunization programs in the US in 2000. Within two years after its introduction, the incidence of IPD among children aged <5 years decreased by 75% [1]. Recent studies have introduced two conjugate vaccines: PCV10 and PCV13. Data suggested that after changing from PCV7 to PCV10, the proportion of serotypes covered would increase to varying degrees in USA, Europe, Africa, and Asia. Changing from PCV10 to PCV13 would further improve the coverage of serotypes by 4% to 7% globally [18]. A study in central Thailand from 2006 to 2009 indicated that PCV7 and PCV13 covered 70.3% and 81.2% of patients aged <5 years, respectively [19]. In Europe, the vaccine serotype coverage ranged from 37% to 100% for PCV7, with mean increases of 7% and 16% for PCV10 and PCV13, respectively [15]. However, data regarding vaccine coverage are rare in China. Among children with pneumonia in five Chinese hospitals, the change from PCV7 to PCV10 increased the vaccine coverage rates by approximately 0.6%, whereas the change from PCV10 to PCV13 increased the vaccine coverage rates by approximately 15.4% [20]. A multicenter study on IPD isolates of children in China [17] showed that PCV7, PCV10, and PCV13 could cover 60.3%, 66.7%, and, 87.8% of all isolates, respectively. In the present study, the coverage rates of PCV7 and PCV10 were same, whereas the vaccine coverage from PCV10 to PCV13 increased by 12.7%. These findings are similar to the results of a previous study conducted by Zhou [21]. Eight (9.2%) isolates with serotype 19A and two (2.3%) isolates with serotype 6A may be responsible for the significant increasing from PCV7 and PCV10 to PCV13. Therefore, PCV13 should be introduced in China because it can protect more pneumococcal diseases in Chinese children.

Pneumococcal infections are treated mainly with antibiotics, especially penicillin. However, the emergence of penicillin-resistant *S. pneumoniae* has been a major challenge. In Asian countries, the incidence rate of penicillin-resistant *S. pneumoniae* in children has increased from 9.1% in 1998 to 1999 [22] to 29.4% in 2000 to 2001 [23]. Before 2000, the rate of penicillin resistance was 10.1% in Japan, 5.2% in the UK, 0.7% in Germany, 24.7% in Spain, 33.9% in France, and 4.9% in Italy [24]. In the present study, only 3 (3.9%) non-meningitis isolates were non-susceptible to penicillin based on the 2010 CLSI criteria. This finding was much lower than that in previous studies because of the change in penicillin breakpoints. When the breakpoint of CLSI 2007 (susceptible when MIC was ≤0.06 µg/mL but resistant when MIC was ≥2 µg/mL) was adopted, the penicillin intermediate rate of the non-meningitis isolates was up to 74.3%, and the resistant rate was 11.5%. Moreover, the resistance rate of penicillin reportedly decreased to 70.6% [25] for children aged <5 years with the use of penicillin oral breakpoints (resistant when MIC was ≥2 µg/mL). Although the resistant rate of *S. pneumoniae* to penicillin decreased dramatically, the tendency of increasing pneumococcal resistance was certain. In the present study, the MIC50 of penicillin in the isolates was 1.0 µg/mL. This value was much higher than that in the non-invasive pneumococci (from 0.032 µg/mL to 0.25 µg/mL) [21], [26] but similar to the results of IPD isolates [17]. Thus, the IPD isolates may have a higher MIC level for penicillin. In other words, to get good clinical outcomes, a higher concentration of antibiotics was required in the treatment of IPD.

In recent years, the resistance rates of *S. pneumoniae* to other β-lactam antibiotics have also increased in China [25]. In the present study, the resistant rates of *S. pneumoniae* to cefaclor and cefuroxime were 81.6% and 79.3%, respectively, which are higher than those in our previous study [17] and in a recent study by Zhao et al. [25]. Ceftriaxone resistance rates were 3.8% and 33.3% in the non-meningitis and meningitis isolates, respectively. In a previous study on the antimicrobial susceptibility of *S. pneumoniae* in eight European countries, the resistance rates to cefotaxime, cefuroxime, and cefpodoxime were only 5.1%, 17.7%, and 17.5%, respectively [27]. Moreover, 56.3% of IPD isolates collected were MDRSP, which is similar to the results of a previous study in Asian countries [28]. This data indicated that the resistance of antibiotics has been a serious problem in China. The inappropriate using of antibiotics may have caused the high resistance rate, and MDR serotypes such as 19F, 19A, 23F, and 14 may have an important effect on this phenomenon. Fortunately, PCV7 and PCV13 covered 75.5% and 85.7% of the MDR pneumococcal isolates, respectively. Thus, they all have potential in controlling MDRSP. Given that serotype 19A plays an important role in China, PCV13 is more useful than PCV7 and the conjugate vaccines could prevent the spread of MDRSP to some extent.

In our study, most of the isolates showed high resistance rate and MIC level to erythromycin (96.6% of isolates with MIC >256 µg/mL), which is similar to the results (96.4%) reported by the Asian Network for Surveillance of Resistant Pathogens (ANSORP) [28]. The two primary mechanisms of macrolide resistance in *S. pneumoniae* are ribosomal methylation and macrolide efflux encoded by the *ermB* and *mefA* genes, respectively. Target-site modification through substitutions in the 23S rRNA and in the L4 and L22 riboproteins is also rare [29], [30]. Macrolide resistance in *Pneumococci* from Asia, Europe, and South Africa is predominantly mediated by the *ermB* gene, which is usually associated with high-level resistance. In North America, the *mefA* gene-encoded efflux shows low-level resistance and accounts for 61% to 85% of macrolide resistance [30]–[32]. In the present study, the mechanism of the *ermB* gene-encoded ribosomal methylation was found to be the primary resistance mechanism in the IPD isolates from Southern China. Furthermore, no significant differences were associated with the serotype and erythromycin resistance levels. Thus, all serotypes showed similar resistance levels to erythromycin. However, erythromycin resistance in Europe is often associated with pneumococcal serotype 14 [15]. In this study, most of the isolates harboring both the *ermB* and *mefA* genes belong to serotypes 19A and 19F, which is consistent with the results of previous studies [33], [34]. However, ANSORP reported that most pneumococcal isolates that contain both the *ermB* and *mefA* genes belong to serotypes 19F and 14 [35]. The results of the present study showed that the prevalence and distribution of serotype 19F or 19A isolates may have contributed to the high rate of macrolide-resistant *Pneumococci* in China.

In summary, the current study provided updated information and changing trends in the antimicrobial resistance and serotype distribution of IPD isolates in Southern China. The data showed an extremely high prevalence of macrolide resistance and an increasing prevalence of MDRSP. Given the high prevalence of resistance and its clinical impact, continuous surveillance of pneumococcal epidemiology is strongly warranted. This study also showed the serotype distribution of *S. pneumoniae* and the coverage of 7-, 10-, and 13-valent conjugate vaccines. Based on our results, the introduction of PCVs, especially PCV13, can reduce pneumococcal disease and the prevalence of MDRSP in China, as what had been achieved in other countries.

The present study also has several limitations. For instance, all the IPD isolates were from one hospital and the situation of antibiotics using was unclear, leading to bias and affecting the results of this study. In addition, only a small sample size of isolates was used in this study. Therefore, isolates from more pediatric patients should be included.
